# Drug–drug interaction analysis based on information bottleneck graph neural network: A review

**DOI:** 10.1097/MD.0000000000042904

**Published:** 2025-06-20

**Authors:** Shuhua Wang

**Affiliations:** a School of Information Engineering, Jingdezhen Ceramic University, Jingdezhen, China.

**Keywords:** drug–drug interaction, graph neural network, information bottleneck, molecule, predictive

## Abstract

The objective of learning drug–drug interactions is to understand the interaction behavior between compound molecules, which has garnered significant interest in the field of compound molecular science due to the potential harm adverse drug interactions may cause to organisms. Existing machine learning methods mostly rely on manually designed representations of compound molecules, overlooking the essence of compound molecules being composed of multiple molecular substructures and constrained by the knowledge of domain experts in the field of compound molecules. In this work, we propose a novel graph neural network framework for learning compound molecule interactions, which investigates the relationship between pairs of compound molecule graphs by detecting core molecular subgraphs of compound molecules. The proposed graph neural network learning framework leverages the fundamental principle of conditional graph information bottleneck to find the minimum information containing molecular subgraph for a given pair of compound molecule graphs. This framework effectively predicts the essence of compound molecule reactions, wherein the core structure of a compound molecule depends on its interaction with other compound molecules. Extensive experiments on common datasets for prediction tasks of compound molecule interactions demonstrate that the proposed graph neural network learning framework enhances the predictive performance of compound molecule interactions.

## 1. Introduction

Drug–drug interaction (DDI) refers to the side effects triggered by the concurrent administration of 2 or more drugs. The side effects are mainly caused by the pharmacological effects or changes in blood drug concentration resulting from the interaction. From 2009 to 2012, 38.1% of adults in the United States took 2 or more drugs, and the rates were 67.2% and 89.8% among those aged 45 and over and those aged 65 and over, respectively.^[1]^ Experts predict that by 2050, people over 65 will account for one-sixth of the global population, leading to an increasing trend of people taking 2 or more drugs. Therefore, it is of great significance to predict compound drug molecular interactions. Traditional manual methods for predicting compound drug molecular interactions still have many limitations. With the rapid development of artificial intelligence technology, more researchers will use AI methods to predict the interactions between compound drug molecules.^[2–5]^ Using deep learning to predict compound drug molecular interactions can eliminate many adverse events caused by drugs.^[6–9]^ Through the research on AI methods for compound drug molecular interactions, the synthesis cost of compound drug molecules can be reduced, the generation process of compound drug molecules can be improved, and the interaction relationships between compound drug molecules can be studied, which is of great significance to both pharmaceutical companies and patients.

Traditional methods for predicting compound drug interactions mainly involve testing metabolic characteristics, analyzing compound drug metabolites,^[10]^ and simulating the binding between drugs.^[11]^ However, the high cost and long experimental time of traditional methods have greatly limited the discovery of potential compound drug interaction relationships. Machine learning has been widely used in predicting compound drug interactions. Based on the assumption that “there is a certain relationship between the probability of compound drug interactions and the similarity of compound drug finger-prints,” Vilar et al^[12]^ proposed the use of interaction profile fingerprint (IPF) to predict compound drug interactions. IPF encodes known drug interaction pairs as binary vectors using 0 and 1, and then generates similarity between drug pairs using a commonly used method to compare the similarity of 2 sets. Finally, the drug interaction matrix is multiplied with the IPF matrix to calculate the probability matrix of interactions. Lu et al^[13]^ proposed a computational framework based on matrix perturbation based on the assumption that “removing edges randomly from the DDI network will not change the eigenvectors of the network’s adjacency matrix.” Gottlieb et al^[14]^ used various attributes including drug substructures, drug side effects, and drug-targets for drug–drug similarity measurement. They calculated multiple features for each pair of drugs and predicted drug interactions using logistic regression. Zhang et al^[15]^ proposed the use of an integrated model that incorporates 8 drug similarity matrices, including drug substructures, drug-targets, drug-enzymes, drug-transporters, drug-pathways, drug-indications, drug side effects, and known drug interaction pairs. They also calculated 6 other drug similarity matrices based on the adjacency matrix of the DDI network. These models were aggregated using 2 integration methods, including weighted average integration rules and classifier integration rules, for DDI prediction.

Prediction methods based on deep learning mostly depend on domain knowledge and manually constructed features in machine learning. With the development and progress of deep learning technology, it can automatically learn abstract features from a large amount of data, and it has strong robustness and generalization ability, which can overcome the limitations existing in the traditional machine learning process. Ryu et al^[16]^ proposed a model named DeepDDI, which combines drug-structure similarity features with Deep Neural Network. This model can predict drug adverse reaction events based on the chemical structures of different drugs, and output them in the form of sentences according to the potential causal mechanism of their interactions. In addition, as long as food structure information is available, it can also be used to predict drug–food interaction (DFI). The prediction of DDI and DFI can provide important information for prescriptions and even dietary advice when taking certain drugs, and can also provide guidance for drug development. Considering that drug-structure similarity features can only provide limited knowledge, coupled with a large number of drug-related features available, Wang et al^[17]^ also proposed to use the Deep Neural Network model to predict potential drug adverse reactions. Different from the DeepDDI model, it considered 3 different types of features at the input end of the model, namely chemical properties, biological properties, and literature information. When analyzing a large number of biomedical literatures, it also considered the relationship between drugs and word vector embeddings. Deng et al^[18]^ also proposed a deep learning framework named DDIMDL that utilizes multiple feature data. It constructs submodels based on deep neural networks using 4 types of drug features including chemical substructures, targets, enzymes, and pathways, and then uses sub-models to learn crossmodal representations between drug pairs. Finally, their model is applied to predict multiple interaction events between drugs. Huang et al^[19]^ proposed the CASTER computing framework. The sequence mining module can effectively describe the functional substructures of drugs. Secondly, by designing the autoencoding module, labeled and unlabeled chemical structure data can be used to improve the prediction accuracy and generalization ability of the model. And by measuring the correlation coefficient between each input substructure and the DDI prediction results in the designed dictionary learning module, the prediction results of DDI can be explained.

Previous research has mostly independently learned features of different drug-related data. With the development of graph neural network technology, the relationships between different drug features can be represented in the form of graph data. This has a positive effect on the interpretable patterns of drug interactions, leading more and more scholars to consider integrating different drug features and using graph neural network methods to study adverse drug reactions.^[20]^ Zitnik et al^[21]^ considered that knowledge of adverse drug reactions between drugs is often limited and may not be observed in relatively small clinical trials. They proposed the Decagon model, a nonlinear multilayer convolutional neural network model for predicting multiple drug interactions based on adverse effect data between drug pairs. The model constructs a multimodal graph composed of protein–protein interaction networks, drug–protein target interaction networks, and drug–drug interaction networks, and introduces additional information as node attribute features. By using graph convolutional neural networks as encoders to embed representations of nodes in the graph, Ma et al^[22]^ considered that the types of adverse effects generated by interactions between 2 drugs are usually nonlinear, and models capturing their relationships are often very complex and lack interpretability. They designed a multi-view autoencoder to predict DDI based on drug side effects, drug-indications, and interactions. In each view, they only consider a specific drug attribute as the drug node feature, and edges between views are constructed based on the similarity between nodes. Attention mechanisms can be added between multiple views, and the weights of each view are determined based on the corresponding task and features. Through nonlinear multi-view fusion, better interpretability and adaptability are obtained. Based on the learned embedding representations, similarities between drugs can be calculated, and the final multi-label classification prediction results can be provided. Deac et al^[23]^ used drug molecular structure information to predict potential drug interactions between drug pairs. For the internal molecular graph, they used a message passing layer to learn embedding representations of each atom based on drug molecular structure graphs containing bonds and atoms. Message passing can provide robust representations for atoms in drugs. To learn a suitable representation of drug pairs, within 2 drugs, atoms within drugs are allowed to learn the representation of the complete drug pair through a shared attention mechanism across drug boundaries. Huang et al^[24]^ proposed the SkipGNN framework, a graph neural network method for predicting molecular interactions. It predicts drug interactions by aggregating information from directly inter-acting neighbors and skipping similarities. Compared to existing graph neural network frameworks, SkipGNN can receive information from two-hop neighbors and direct neighbors in the interaction network and transform them nonlinearly to obtain useful prediction information.

Predictive methods based on knowledge graph (KG) is a heterogeneous structure that stores facts about the world in the form of a machine-readable graph. The nodes and relationships in the graph are represented as triplets, where nodes represent entities existing in the objective world, and edges represent factual relationships between entities. Facts are modeled in the form of triplets (subject, predicate, object), such as (aspirin, drug-target, Cyclooxygenase-1), where the subject entity (drug aspirin) is connected to the object entity (target protein Cyclooxygenase-1) through a predicate relationship (drug-target). Since the proposal of KG, it has attracted widespread attention in many research fields, such as recommendation systems,^[25,26]^ and question answering systems.^[27,28]^ In recent years, there has been a considerable amount of research in the field of biomedicine based on KGs.^[29–31]^ For the DDI prediction task, Celebi et al^[32]^ applied RDF2Vec, TransE, and TransD methods separately in the DrugBank KG, trained node representations in the KG, and evaluated the DDI prediction performance using methods such as random forests, logistic regression, and naive Bayes. Building upon Celebi work, Karim et al^[33]^ integrated multiple data sources into a large KG, trained it using 6 different KG embedding methods, and selected the best-performing KG embedding method ComplEx to learn representations of nodes in the KG. Finally, they proposed the Conv-LSTM framework for binary prediction of drug pairs. Mohamed et al^[34,35]^ proposed a new tensor decomposition-based KG embedding method TriModel, which extends the DisMult and ComplEx models. They generated a KG related to drugs and targets using existing data and trained the model to learn effective vector representations of drugs and targets in the KG. In the training process, TriModel represents both node and relationship vectors using 3 embedding vectors. It uses embedding interaction functions, including 1 symmetric interaction and 2 asymmetric interactions, when calculating the scoring function. This allows the prediction of drug-target models to be generalized to drug interaction prediction tasks. Considering that existing KG methods are based on directly learning the latent embedding representations of nodes without capturing rich neighborhood information of each entity in the KG, Lin et al^[35]^ proposed the KGNN model. By selectively aggregating neighborhood information on the KG, it can learn the high-order structure and semantic relationships of the KG, and generate drug entity representations with rich information to predict DDI. Considering that KGs are too large and contain some noise, i.e., among the thousands of nodes, there are millions of edges, but only a small subgraph is relevant to the current predicted drug pair, Yu et al^[36]^ proposed the SumGNN model. This model can effectively locate subgraphs relevant to drug interactions through a subgraph extraction module, and then use self-attention mechanism methods based on subgraphs to generate inference paths for drug interactions within subgraphs. Furthermore, through a multichannel knowledge and data integration module, the model can utilize a large amount of external biomedical knowledge to improve the performance of multi-class DDI prediction. Zhu et al^[37]^ proposed a DTA prediction method with interactive learning and automatic encoding mechanism. A variational autoencoder system with attention model and convolutional neural network cascade structure was constructed using mutual transformer-drug target affinity. Established feature expression relationships for each substructure in a single molecular sequence, increased information exchange pathways between molecular sequence pairs, and supplemented the expression of correlations between molecular substructures. Zhu et al^[38]^ proposed a graph optimization module based on diffusion model to improve the representation of molecular graph structure and enhance the interpretability of graph representation while obtaining the best feature representation. Zhu et al^[39]^ proposed a DTA prediction model multi-scale diffusion and interactive learning that utilizes multi-scale diffusion and interactive learning. To address the limitations of current methods, a multi-scale graph diffusion convolution module has been introduced, which can effectively capture the complex interactions between nodes in drug molecule graphs. Utilizing CNN Transformer Network blocks to capture the interactions and dependencies between different amino acids, thereby enhancing the model’s representation and learning capabilities.

In summary, there are many methods currently available for predicting drug interactions. Current methods based on drug interactions can be broadly classified into 2 categories: traditional machine learning DDI prediction methods and deep learning DDI prediction methods. With the accumulation of a large amount of biomedical data and the increase in available data, the application of deep learning methods in the field of biomedicine is becoming more widespread. The development of graph neural network technology and the increase in knowledge of attributes related to drugs allow graph neural network-based methods to provide more semantic information for drug representations in DDI prediction and to provide some interpretability for the mechanism of drug interactions, attracting more and more attention from scholars in the field of graph neural networks.

This paper proposes a new graph neural network framework for learning the interactions between compound molecules. Using the fundamental principle of conditional graph information bottleneck (IB), it finds a molecular subgraph containing the minimum information for a given pair of compound molecular graphs. The framework effectively predicts the essence of compound molecular reactions, where the core structure of a compound molecule depends on its interactions with other compound molecules. Predicting the interaction of complex drug molecules can effectively avoid the potential harm to the organism caused by adverse interactions that may occur when taking more than 2 drugs. In addition, by predicting the interaction between complex drug molecules, it can reduce the synthesis cost of complex drug molecules and improve the generation process of complex drug molecules, which is of great significance to pharmaceutical companies and patients.

## 2. Related knowledge

### Definition 3.1:

Mutual information: I(X;Y) expressed as mutual information X and Y 2 variables:


$$\begin{array}{l} I(X;Y)=\int_{X}\int_{Y}p(x,y)\log\frac{p(x,y)}{p(x)\;p(y)}dxdy. \end{array}$$

### Definition 3.2:

IB^[37]^: suppose there are 2 variables X and Y, both random variables, IB by compressing X to bottleneck T while maintaining useful information related to label Y:


$$\begin{array}{l} \underset{T}{\min}-I(Y;T)+\beta I(X;T). \end{array}$$

Where β is the Lagrange multiplier that balances X and Y.

### Definition 3.3:

Optimal graph: suppose that the compound molecule graph G and its label value Y are input, and the optimal graph $G_{IB}$ output after passing through the graph neural network model of the IB is:


$$\begin{array}{l} G_{IB}=\arg\underset{G_{IB}}{\min}-I(Y;G_{IB})+\beta I(G;G_{IB}). \end{array}$$

The graph neural network model of IB determines the relationship between the compound molecular graph and the label value by maximizing term $I(Y;G_{IB})$ and optimizing term $I(G;G_{IB})$. Where, $X_{IB}$ represents the feature set of compound molecules in molecular graph G, and $A_{IB}$ represents the adjacency matrix of compound molecules in molecular graph G.^[38,39]^

Lemma 3.4^[37]^: Given the compound molecular graph pair $G^{x}$, $G^{y}$ and the label value Y, assuming that $G_{\varepsilon }^{x}$ is the noise in the compound molecular diagram $G^{x}$ that has nothing to do with the prediction of the graph neural network model, then:


$$\begin{array}{l} I(G_{IB}^{x};G_{\varepsilon }^{x}|G^{y})\leq -I(Y;G_{IB}^{x}|G^{y})+I(G^{x};G_{IB}^{x}|G^{y}). \end{array}$$

The proof of the above is as follows:


$$\begin{array}{l} I(G^{x};G_{IB}^{x}|G^{y})=I(G_{IB}^{x};G^{x},G^{y})-I(G_{IB}^{x};G^{y}) \end{array}$$

$$\begin{array}{l} \geq I(G_{IB}^{x};Y,G_{n}^{x},G^{y})-I(G_{IB}^{x};G^{y})=I(G_{IB}^{x};G_{n}^{x},G^{y})+I(G_{IB}^{x};Y|G_{n}^{x},G^{y})-I(G_{IB}^{x};G^{y}) \end{array}$$

$$\begin{array}{l} =I(G_{IB}^{x};|G_{n}^{x}|G^{y})+I(G_{IB}^{x};Y|G_{n}^{x},G^{y}) \end{array}$$

Assume that Y and $G_{n}^{x}$, $G^{y}$ and $G_{n}^{x}$, Y and variable $\left(G_{n}^{x},G^{y}\right)$ are all independent, then:


$$\begin{array}{l} I(G_{IB}^{x};Y|G_{n}^{x},G^{y})=H(Y|G_{n}^{x},G^{y})-H(Y|G_{n}^{x},G_{IB}^{x},G^{y}) \end{array}$$

$$\begin{array}{l} \geq H(Y|G^{y})-H(Y|G_{IB}^{x},G^{y})=I(Y;G_{IB}^{x},G^{y}) \end{array}$$

This section introduces the concepts related to variable mutual information and the principles related to IBs. Among them, the IB principle is the core method in the graph neural network model proposed in this article.

## 3. Graph neural network model

The paper proposes a novel framework for learning relationships between compound molecules based on graph neural networks: compound molecules are composed of multiple molecular subgraphs. Recently, the IB theory^[40]^ has been applied to learning important subgraphs of input graphs in interpretable graph neural networks^[41,42]^ utilize the principle of IB to determine which parts of the compound molecule graphs data to retain and which to discard. By incorporating the IB principle into the attention mechanism of graph neural networks, the attention mechanism of graph neural networks is used to determine which edges of molecular graphs to retain, generating a graph that retains interpretable compound molecule subgraphs. This paper proposes a new Graph Neural Network (IBGCN), which is an effective model for learning relationships between molecular graph data, by analyzing compound molecular subgraph data to predict the relationship between pairs of compound molecular graph data. Due to its design for learning relationships in compound molecular graph data, IBGCN captures important molecular subgraphs that vary according to the original compound graphs. Given a compound molecular graph $G^{x}$, IBGCN learns the significant subgraph, maximizing the mutual information between the compound graph and the graph neural network model’s response, while minimizing the interaction between the compound graph and the significant subgraph. The core idea of this approach, termed Graph IB, is described as follows:

Suppose that given a set of chemical molecular graphs G, the type of interaction of these chemical molecules is $I=\{I_{i}{\}}_{i=1}^{m}$, a *n*-dimensional chemical molecular data set DDI $D=\{{\left(G^{x},G^{y},r\right)}_{i}{\}}_{i=1}^{n}$, where $G^{x}$ and $G^{y}$ represents the interaction type of a pair of chemical molecules, to find a graph neural network model and analyze the probability of the combination of any 2 chemical molecular graphs will lead to a given interaction type. Where $G^{x}$ and $G^{y}$ represent a pair of chemical molecule interaction type r type $I_{i}$, we need to find a graph neural network model fG×G×I→[0,1] to analyze the probability that any combination of 2 chemical molecule graphs will lead to a given interaction type $I_{i}$. The model takes a chemical molecule data set DDI tuple $\left(G^{x},G^{y},r\right)$ as input. Chemical molecule graphs $G^{x}$ and $G^{y}$ are both represented as graph G=(V,E). G represents a compound molecular graph, where $V=\{v_{1},v_{2},\cdots ,v_{n}\}$ represents the set of nodes in the compound molecular graph, and $E=\{e_{1},e_{2},\cdots ,e_{m}\}$ represents the compound molecular graph. Collection of edges. The adjacency matrix of compound molecule graph G is $A\in R^{n\times n}$, and the characteristic matrix of compound molecule graph is $X\in R^{n\times F}$, where F is the number of features of the graph. Adjacency matrix $A_{ij}=1$ means that compound molecule node i is connected to compound molecule node j, and $A_{ij}=0$ means that compound molecule node i is not connected to compound molecule node j. Chemical molecule graph G simply represents the molecular graph of the medicinal chemical molecular structure, in which each molecular graph node $v_{i}$ represents a drug atom, and the eigenvectors $h_{i}\in R^{F}$ and $\left(v_{s},v_{t}\right)$ of the medicinal chemical molecular structure represent the existence of chemical bonds between nodes $v_{s}$ and $v_{t}$ of each molecular graph. Each molecular graph node $v_{i}$ represents a drug atom, and the eigenvectors $h_{i}\in R^{F}$, and $\left(v_{s},v_{t}\right)$ of the drug chemical molecular structure represent the existence of chemical bonds between nodes $v_{s}$ and $v_{t}$ of each molecular graph. Each type of molecular graph interaction $I_{r}\in I$ is represented as a learnable matrix $M_{r}\in R^{n\times d}$ in the network model.

In the graph neural network proposed in this article, the multilayer (Graph Attention Networks) layer of the graph neural network^[28]^ is used as the update of molecular graph node features. In order to calculate the eigenvector of molecular graph node i in the l+1 graph neural network layer: ${h_{s}}^{\left(l+1\right)}=W^{\left(l+1\right)}{h_{s}}^{l},s=1,2,\cdots ,n$, Among them, matrix $W^{\left(l+1\right)}\in R^{F^{\prime }\times F}$ is the learnable matrix from the l graph neural network layer to the l+1 graph neural network layer of the model.

The correlation between each molecular node in the neighborhood of the molecular graph node is calculated using the attention mechanism of the graph neural network model. In the graph neural network model, not all molecule nodes are equally important when updating the feature vector of the molecule node, so each molecule node is assigned a learnable correlation variable. Calculate the correlation between molecule node j and molecule node i as follows:


$$\begin{array}{l} \alpha_{ij}=\frac{\exp(LeakyReLU(e_{ij}))}{\sum_{s\in N_{i}⋃\{i\}}\exp(LeakyReLU(e_{is}))}. \end{array}$$

Where LeakyReLU is an activation function to alleviate the problem of zero gradient, and $e_{ij}$ is the weight vector: $e_{ij}=a^{T}\left[{\hat{h}}_{i}^{l+1}x2016;{\hat{h}}_{i}^{l+1}\right]$. Then the characteristics of molecule node i in the l+1 layer are calculated by learning the molecule nodes in model aggregation $N_{\left(i\right)}⋃\{i\}$ as: zil+1=∑s∈Ni⋃{i}αish^sl+1.

Then calculate the eigenvector of compound molecule node i: ${h_{i}}^{\left(l+1\right)}=\sigma (z_{i}^{l+1})$.The IBGCN model proposed in this article uses a K -head graph neural network attention mechanism, in which the graph neural network layer performs the following K linear transformation on the compound molecule nodes: $h_{i}^{l+1}=\sigma (x2016{;}_{k=1}^{K}{\left[z_{i}^{k}\right]}^{l+1})$. In order to improve the convergence speed of the IBGCN model proposed in this article, normalization is performed after the graph neural network layer in each model: ${h_{i}}^{\left(l+1\right)}=\sigma (LeakyReLU(z_{i}^{l+1}))$.

In compound molecular structure extraction, the IBGCN model collects subgraphs of different compound molecules. Each compound subgraph is centered on the molecular node, and its corresponding molecular feature vector $z^{l+1}$ is the subgraph feature vector formed by molecular node $s\in N_{i}⋃\{i\}$. In the graph neural network layer in the IBGCN model, the information of all compound molecular subgraphs is aggregated, and each compound molecular subgraph is weighted by a corresponding importance coefficient of β. Compound molecular graph $G^{x}$ aggregates the characteristic information of all molecular subgraphs in the l layer, represented as ${g_{x}}^{l}=\sum_{i=1}^{n}\beta_{i}z_{i}^{l}$.

In the IBGCN model, the interaction mapping between compound molecular graphs $G^{x}$ and $G^{y}$ is defined as $I_{ij}=sim(E_{i}^{x},E_{j}^{y})$, where $I\in R^{n_{x}\times n_{y}}$, sim(.,.) represent the cosine similarity between variables, $E^{x}\in R^{n_{x}\times d}$ and $E^{y}\in R^{n_{y}\times d}$ are the molecular node matrices of compound molecular graphs $G^{x}$ and $G^{y}$ respectively, $n_{x}$ and $n_{y}$ represents the number of molecular nodes in compound molecular graphs $G^{x}$ and $G^{y}$ respectively. Then, the pairing graph based on compound molecules is defined as: $\tilde{E^{x}}=I\cdot E^{y}$, $\tilde{E^{y}}=I\cdot E^{x}$. $\tilde{E^{x}}$ represents the interactive relationship between the nodes in the molecule of compound molecular graph $G^{x}$ and the molecular nodes in compound molecular graph $G^{y}$. Use $\tilde{E^{x}}$ and $E^{x}$ to calculate the final molecular node feature matrix of compound molecular graph $G^{x}$: $H^{x}=(E^{x}x2016;\tilde{E^{x}})\in R^{nx\times 2d}$.

Use $\tilde{E^{y}}$ and $E^{y}$ to calculate the final molecular node feature matrix of compound molecular graph $G^{y}$: $H^{y}=(E^{y}x2016;\tilde{E^{y}})\in R^{ny\times 2d}$. In the IBGCN model, the objective function for optimizing the graph neural network model is: $\min-I(Y;G_{IB}^{x}|G^{y})+\beta I(G^{x};G_{IB}^{x}|G^{y})$.

After obtaining the molecular subgraph information ${g_{x}}^{l}$ and ${g_{y}}^{l}$ of the input compound molecular figures $G^{x}$ and $G^{y}$ at the graph neural network layer, the IBGCN model uses the common attention mechanism^[33]^ of the graph neural network to explain the importance between the structures of the molecular subgraphs $G^{x}$ and $G^{y}$:


$$\begin{array}{l} \gamma_{ij}=b^{T}\tanh(W_{x}{g_{x}}^{l}+W_{y}{g_{y}}^{l}),i=1,2,\cdots ,L,j=1,2,\cdots ,L. \end{array}$$

Where $W_{x}$ and $W_{y}$ are representative weight matrices.

The prediction probability of the interaction between molecular subfigures $G^{x}$ and $G^{y}$ is:


$$\begin{array}{l} P(G^{x},G^{y},r)=\sigma (\sum_{i}\sum_{j}\gamma_{ij}{\left({g_{x}}^{l}\right)}^{T}M_{r}{g_{y}}^{l}). \end{array}$$

The loss function of sample ${\left(G^{x},G^{y},r\right)}_{i}$ of the dataset in the IBGCN model is:


$$\begin{array}{l} L=\frac{1}{N}\sum_{i=1}^{N}\left(\log(p_{i})+\log(-p_{i}^{\prime })\right). \end{array}$$

Based on the principle of IB, by replacing non-important nodes with noise, the information of $G^{x}$ and $G^{y}$ is compressed to a minimum, with the following upper bound for minimization:


$$\begin{array}{l} I(G_{IB}^{x};G^{x},G^{y})\leq E_{G^{x},G^{y}}{\left[(\frac{1}{2n_{x}}-\frac{1}{2})\log\sum_{j=1}^{n_{x}}(1-\lambda_{i}\right)}^{2}+\frac{1}{2n_{x}}{\left(\frac{\sum_{j=1}^{n_{x}}(h_{j}^{x}-\mu_{h^{x}})}{\sigma_{h^{x}}}\right)}^{2}] \end{array}$$

The proof of the above is as follows:

According to the principle of KL divergence, we have:


$$\begin{array}{l} I(G_{IB}^{x};G^{x},G^{y})\leq E_{G^{x},G^{y}}[KL(p_{\phi }(z_{G_{IB}^{x}}|G^{x},G^{y})∥q(z_{G_{IB}^{x}}))] \end{array}$$

where $q(z_{G_{IB}^{x}})=N(N^{x}\mu_{h^{x}},N^{x}\sigma_{h^{x}}^{2})$, $p_{\phi }(z_{G_{IB}^{x}}|G^{x},G^{y})=$$N(N^{x}\mu_{h^{x}}+\sum_{j=1}^{n_{x}}\lambda_{j}\mu_{h^{x}},$$\sum_{j=1}^{n_{x}}{\left(1-\lambda_{j}\right)}^{2}\sigma_{h^{x}}^{2})$, so:


$$\begin{array}{l} I(G_{IB}^{x};G^{x},G^{y})\leq E_{G^{x},G^{y}}{\left[(\frac{1}{2n_{x}}-\frac{1}{2})\log\sum_{j=1}^{n_{x}}(1-\lambda_{i}\right)}^{2}+\frac{1}{2n_{x}}{\left(\frac{\sum_{j=1}^{n_{x}}(h_{j}^{x}-\mu_{h^{x}})}{\sigma_{h^{x}}}\right)}^{2}]+C \end{array}$$

where C is ignored during optimization.

## 4. Discussion

Task: interactive learning between drug molecular maps. Given a set of drug molecular graph pair $D=\{(G_{i}^{x},G_{i}^{y})\},i=1,2,\cdots ,n$ and drug molecular graph pair label $Y=\{Y_{i}\},i=1,2,\cdots ,n$, a graph neural network model IBGCN based on IB is trained to predict whether the relationship value of drug molecular graph pair is equal to the label value, that is $Y_{i}=M(G_{i}^{x},G_{i}^{y})$.

Hardware configuration: The IBGCN model is implemented in PyTorch Geometric. All experiments were run on Windows 11 OS with a NVIDIA GeForce GPU (RTX 3080), the CPU processor (11th Gen Intel(R) Core(TM) i7-11700F @ 2.50GHz) and 32.0 GB of RAM.

Method Comparison: In each task, our IBGCN model is compared with other relevant models as follows:

- GCN^[2]^: a graph convolutional network that uses the average of neighboring node features as convolutional weights and learns node representations through multiple convolutional layers;
- GIN^[9]^: a graph isomorphism network that parameterizes a generic injective neighborhood aggregation function with neural networks, possessing injective properties on the aggregation function for neighbors;
- MPNN^[31]^: each node in a compound molecule has a hidden state. For each node, the hidden states of all neighboring nodes and possible edges are aggregated together with the node itself, and then used to update the node’s hidden state using the obtained messages and the node’s previous hidden state;
- SSI-DDI^[20]^: substructure-similarity interaction for DDI. It represents the hidden representations of each node in compound molecules as substructures and then calculates the interactions between these substructures to predict DDI.

The datasets used in this article are as follows:

- DeepDDI: DeepDDI dataset is a dataset used for predicting DDI and DFI. This dataset contains 192303 DDIs, involving a total of 99 types.
- ZhangDDI: ZhangDDI dataset is a dataset used for predicting DDI. This dataset contains 48,548 pairwise interaction data and 548 drugs.
- ChChMiner: ChChMiner dataset is a dataset used for predicting DDI. This dataset contains 48,514 labeled DDIs and 1322 drugs.

In this paper, area under the receiver operating characteristic curve (AUROC), F1 score, and accuracy are used to evaluate the experimental results of different approaches on the ChChminer dataset, as shown in Table 1. It can be observed from Table 1 that the IBGCN model outperforms other models in all 3 metrics: AUROC, F1 score, and accuracy, with a superiority margin of 3.22% in terms of accuracy.

**Table 1:** Comparison evaluation of different approaches on the ChChminer dataset.

	AUROC	F1	ACCURACY
GCN	94.58	87.06	87.38
GIN	97.42	91.78	92.01
MPNN	96.86	91.26	91.63
SSI-DDI	98.22	93.27	93.96
IBGCN	99.59	95.52	97.18

AUROC = area under the receiver operating characteristic curve, DDI = drug–drug interaction, F1 = F1 score.

Using AUROC, F1 score, and accuracy, the experimental results of the IBGCN model on different datasets are evaluated, as shown in Table 2. It can be seen from Table 2 that the IBGCN model performs better on the ChChminer and DeepDDI datasets compared to the results on the ZhangDDI dataset, particularly with a superiority margin of 11.17% in terms of accuracy.

**Table 2:** Comparison evaluation of the IBGCN model on different datasets.

	AUROC	F1	ACCURACY
ChChminer	99.59	95.52	97.18
DeepDDI	99.24	96.37	97.56
ZhangDDI	97.03	91.78	86.01

AUROC = area under the receiver operating characteristic curve, DDI = drug–drug interaction, F1 = F1 score.

Using AUROC, F1 score, and accuracy, the experimental results of the IBGCN model on the ChChminer dataset are evaluated, as shown in Table 3. It can be observed from Table 3 that the performance of the IBGCN model on the training, testing, and validation sets fluctuates moderately, indicating a balanced performance across these sets.

**Table 3:** Comparison evaluation of the IBGCN approach on training, testing, and validation sets.

	AUROC	F1	ACCURACY
ChChminer	99.59	95.52	97.18
DeepDDI	99.24	96.37	97.56
ZhangDDI	97.03	91.78	86.01

AUROC = area under the receiver operating characteristic curve, DDI = drug–drug interaction, F1 = F1 score.

**Figure 1.**
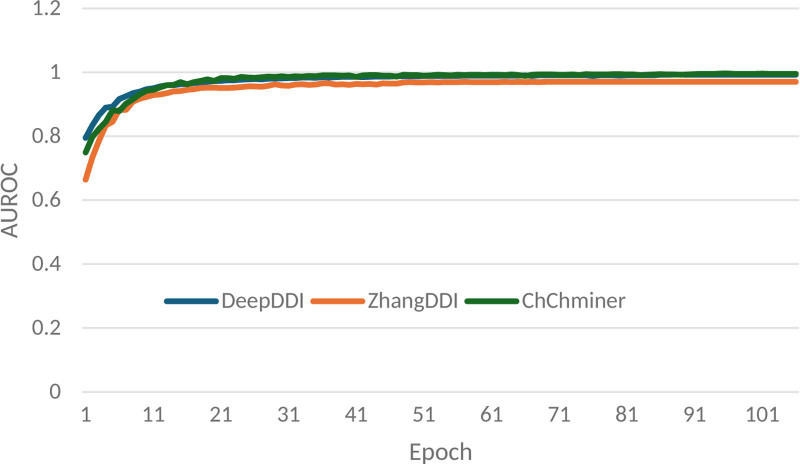
Variation curve of epochs and AUROC for the IBGCN approach on different datasets. AUROC = area under the receiver operating characteristic curve.

The variation trend of AUROC with increasing epochs for the IBGCN model on different datasets is evaluated, as depicted in Figure 1. It can be seen from Figure 1 that AUROC fluctuates significantly when the number of epochs is small, but tends to stabilize around 20 epochs.

**Figure 2.**
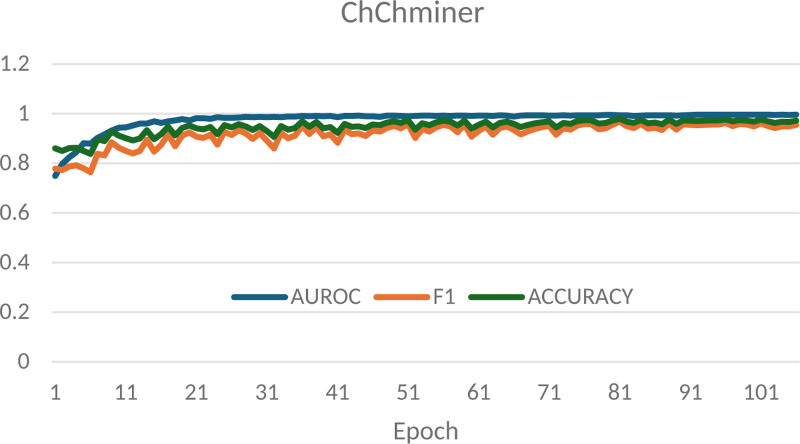
Variation curve of epochs and evaluation metrics on the ChChminer dataset.

The variation curves of AUROC, F1 score, and accuracy with increasing epochs for the IBGCN model on the ChChminer dataset are shown in Figure 2. It can be observed from Figure 2 that all metrics exhibit minor fluctuations across different epochs.

## 5. Conclusions

This paper proposes a novel graph neural network model, IBGCN, for DDI prediction tasks. The IBGCN model predicts the interaction behavior between drug molecules by detecting core subgraphs in drug molecule pairs. The main principle is to identify core substructures in drug molecules, which have a genuine causal relationship for DDI prediction within a new conditional intervention framework. Given a pair of drug chemical molecules, based on the bottleneck principle of drug chemical molecule graph information, the core substructures of the given drug chemical molecules are found, containing the maximum relevant information for the DDI prediction task. Extensive experiments on different datasets demonstrate that the proposed IBGCN model a outperforms other models in all 3 metrics: AUROC, F1 score, and accuracy, with a superiority margin of 3.22% in terms of accuracy. The IBGCN model performs better on the ChChminer and DeepDDI datasets compared to the results on the ZhangDDI dataset.

## Author contributions

**Conceptualization:** Shuhua Wang.

**Data curation:** Shuhua Wang.

**Formal analysis:** Shuhua Wang.

**Resources:** Shuhua Wang.

**Writing – original draft:** Shuhua Wang.

**Writing – review & editing:** Shuhua Wang.
